# Role of the cAMP-PKA-NF-κB pathway in Mucin1 over-expression in A549 cells during Respiratory syncytial virus infection

**DOI:** 10.1186/s12879-023-08837-1

**Published:** 2023-11-30

**Authors:** Yingkang Jin, Dongwei Zhang, Kuimiao Deng, Peiqiong Wu, Diyuan Yang, Zhiwei Xie, Wenjun Qiu, Guangyuan Yu

**Affiliations:** Department of Respiratory Medicine, Guangzhou Women and Children’s Medical Center, Guangzhou Medical University, Guangzhou, 510623 Guangdong China

**Keywords:** Respiratory syncytial virus, Mucin 1, cAMP, Protein kinase A, NF-κB

## Abstract

Respiratory syncytial virus (RSV) is the most common pathogen associated with acute lower respiratory tract infections in infants and young children worldwide. RSV commonly presents as bronchiolitis in young children; however, it can sometimes progress to pneumonia, respiratory failure, apnoea and even death. Although mucin1 (MUC1), a type of transmembrane glycoprotein present on airway epithelial surfaces, plays a crucial anti-inflammatory role in airway infections; however, its roles in RSV-associated acute lower respiratory tract infections have rarely been explored. In this study, we first revealed very high MUC1 protein levels in the exacerbation phase in sputum samples from children with RSV bronchiolitis. Because MUC1 is the downstream target of tumour necrosis factor-alpha (TNF-α) in RSV-infected A549 cells, we observed the inhibition of NF-κB activity, main downstream signalling of TNF-α and remarkably reduced levels of MUC1 in RSV-infected and TNF-α treated A549 cells. Furthermore, the cyclic adenosine monophosphate (cAMP) analogue (dbcAMP) downregulated the protein levels of p-IκBα and MUC1 in TNF-α-treated A549 cells. By contrast, a protein kinase A inhibitor (KT5720) up-regulated the levels of those proteins. dbcAMP and KT5720 had the same effects on MUC1 protein levels in RSV-infected A549 cells. In conclusion, we found that the cAMP-PKA-NF-κB pathway may play a role in the regulation of MUC-1 over-expression during RSV infection.

**Supplementary Information:**

The online version contains supplementary material available at 10.1186/s12879-023-08837-1.

## Introduction

Respiratory syncytial virus (RSV) is the most common pathogen associated with acute lower respiratory tract infections (ALRIs) in young children worldwide [1]. Almost all children become infected with RSV by age 2 years, and more than 68% of children become infected by their first birthday [2]. The WHO has estimated that the annual burden of RSV-associated ALRIs was approximately 3.4 million and that the annual in-hospital deaths were approximately 265,000 worldwidely [3]. Yet, RSV, an enveloped RNA virus that is the most prevalent viral pathogen that was discovered over 50 years ago, is far from being well known [4]. Currently, besides supportive care, no curative therapy or useful vaccine is available for treating children RSV infections [5]. Hence, further exploration of the underlying molecular mechanisms of RSV-induced pathologies is particularly significant for the establishment of new treatment strategies.

Mucin 1 (MUC1), a transmembrane epithelial glycoprotein, has been shown to function as a physical barrier in limiting pathogen infection and colonisation and exerts anti-pathogenic activities by suppressing inflammatory responses [6]. Compared with wild-type (WT) mice, MUC1 knockout mice showed increased airway inflammation during infection with *P. aeruginosa* [7]. MUC1 − / − mice likely showed higher *Pneumococcal* loads and severe airway inflammation during infection with *Streptococcus pneumoniae*, as compared to WT mice [8]. In terms of viral infections, McAuley JL et al. reported that compared with WT mice, MUC1 − / − mice showed enhanced morbidity and mortality rates and heightened airway inflammation during infection with influenza A [9]. Particularly, Li Y et al. found that RSV could up-regulate MUC1 expression in A549 epithelial cells in a time-dependent manner and that MUC1 could suppress RSV-induced inflammatory responses in A549 epithelial cells [10]. However, the exact role of MUC1 during RSV infection has not yet been elucidated in clinical settings [6].

MUC1 plays an anti-inflammatory role in many infections and chronic inflammatory lung diseases. For instance, Zheng Z et al.found that MUC1 expression was increased in the sputum of patients with chronic obstructive pulmonary disease during the acute exacerbation phase [11]. Mavi AK et al. found that the serum MUC1 protein levels were up-regulated in 88.2% of patients with pigeon-sensitive asthma [12]. Nevertheless, MUC1 expression patterns have never been investigated in children with RSV-associated ALRIs. Tumour necrosis factor alpha (TNF-α) is the key regulator of MUC1 expression in the pathogenic infection of airway epithelial cells [13]. In RSV-infected A549 cells, increased MUC1 levels are preceded by increased TNF-α production, after which MUC1 suppresses a further increase in the TNF-α levels, generating a negative feedback loop to limit RSV-induced inflammation [10]. Anti-tumour necrosis factor receptor 1 antibody significantly blocked the increasing MUC1 levels induced by TNF-α [14]. However, there is no report on how TNF-α induced MUC1 over-expression in A549 cells after RSV infection. Because TNF-α can up-regulate the levels of mucus protein (MUC5AC) in airway epithelial cells dependent on Nuclear factor kappa-B (NF-κB) activation, we explored the role of the NF-κB signalling pathway in TNF-α induced MUC1 expression during RSV infection [15, 16]. Because the Cyclic Adenosine monophosphate/protein kinase A (cAMP/PKA) pathway not only plays a crucial role in RSV pathogenesis [17, 18] but also inhibits NF-κB transcription activity [19], we explored its role in MUC1 over-expression.

Taken together, we determined the protein levels of MUC1 and TNF-α in the sputum of children with RSV-associated ALRIs and explored the role of the cAMP/PKA/NF-κB signalling pathway in TNF-α-induced MUC1 over-expression during RSV infection using A549 cells.

## Methods and reagents

### Patients and sample collection

The study protocol was approved by the ethics committee of the Guangzhou Women and Children's Medical Center. All patient guardians signed a written informed consent form for participation in the study. Twenty-five hospitalised patients (age range, 3–9 months; median age, 5 months; boy:girl ratio, 14:11) who were diagnosed with RSV bronchiolitis and met the characteristic exacerbation period and clinical manifestations (acute respiratory illness with difficult breathing, wheezing, rales, during the first day of hospitalization.) and whose etiological agent was RSV, as detected through the RSV IgM viral antigen in serum or using RSV PCR with nasopharynx aspirate samples, were enrolled. Patients infected with other respiratory viral pathogens, such as rhinovirus, metapneumovirus, adenovirus and flu virus, were excluded. All samples were collected from October 2019 to February 2020. Deep sputum samples were collected in pairs, and the MUC1, TNF-α, IL-6, IL-8 and IL-1β levels in the exacerbation and recovery stages (significant relief of dyspnoea, no supplemented oxygen and no significant rales, during the day before discharge from the hospital) were detected using ELISA. The relationship between the MUC1 and TNF-α protein levels was assessed using Person’s correlation coefficient analysis.

### Collection of induced sputum

Fresh lower respiratory sputum samples were collected from each patient using a negative pressure suction tube; the samples were induced by atomising with 3% saline solution for 15 min. Each sample was immediately treated with a fixed proportion of 0.1% dithiothreitol (AMRESCO, USA) and then fixed with Dulbecco's phosphate-buffered saline and stored at 4 °C overnight. Next, the supernatant was separated from the cell pellet in the sputum lysate buffer by centrifugation (800 g for 10 min at 4 °C) and stored at − 80 °C until further use.

### ELISA

The supernatant of the induced sputum was used to assess the MUC1 protein levels using direct ELISA. First, 96-well plates were coated overnight at 4 °C with the diluted sputum samples (0.05 mM bicarbonate buffer, pH 9.5, 1:1000), blocked with 3%BSA dissolved in PBS and then incubated with a mouse monoclonal antibody against MUC1 (ab45167, Abcam, UK) (1:250 diluted in blocking buffer) for detecting the MUC1 protein levels [11].

The concentrations of TNF-α, IL-6, IL-8 and IL-1β in the sputum samples were assessed using ELISA kits (DTA00D, D6050, D8000C, DLB50, R&D Systems, USA), according to the manufacturer's instructions.

### Cell culture

A549 human alveolar adenocarcinoma or Hep2 cells were all were cultured in Dulbecco's modified Eagle's medium (DMEM) supplemented with 10% foetal bovine serum, 100 U/ml penicillin and 100 μg/ml streptomycin and incubated at 37 °C and in a humidified atmosphere containing 5% CO_2_.

### Viral amplification and cell infection

The RSV A2 standard strain and the Hep-2 and A549 cell lines were provided by the Guangzhou Institute of Respiratory Health. For virus preparation and titre measurement, Hep-2 cells were cultured in T75 cell culture flask. When the cells had grown into a confluent monolayer, a virus solution containing stock RSV prepared in DMEM culture medium was added. After 2 h of adsorption, the virus solution was discarded and fresh culture medium was added. The cells were cultured for 5 more days. The RSV virus was harvested when > 70% of the cells developed pathological changes. The culture mediums were centrifuged at 3000 rpm for 10 min, then the supernatants were purified by sucrose density gradient centrifugation using the HBSS buffer containing the 35% sucrose. The plaque assay was used to determine the viral titer, and the viral titer after amplification was 1.4*10^6^ PFU/ml.

The A549 cells were seeded into 6-well plates at a density of 1 × 10^5^ cells per well. For viral infection and (or) drug treatment, the A549 cells in the RSV infection group (RSV group) were infected with RSV virus solution (multiplicity of infection [MOI] = 1). The culture supernatant from the uninfected Hep-2 cells was added to the control group. Recombinant human TNF-α protein were purchased from R&D Systems (210-TA-020, Minneapolis, USA). All compounds (Bay-11–7082, dbcAMP, KT5720) were purchased from Sigma-Aldrich (196,870, 28,745-M, 420,320, St. Louis, USA).

### Preparation of protein extracts

The A549 cells were subjected to various treatments and then washed with PBS before being dislodged with a cell scraper. Nuclear protein extracts were prepared using the NE-PER Nuclear and Cytoplasmic Extraction Reagent, according to the manufacturer's protocol (78,835, Thermo Fisher Scientific, USA). The cultured cells were lysed in radioimmunoprecipitation assay lysis buffer containing 1% protease inhibitor cocktail (R0278, P8340, Sigma-Aldrich, USA), 5 μM EDTA and 200 μM 4-(2-aminoethyl) benzenesulfonyl fluoride hydrochloride to extract the total protein.

### Western blotting

BCA (Bicinchonic Acid) Protein Quantitative Kits were used for determining the concentration of proteins extracted(BCA1, Sigma-Aldrich, USA). Equal amounts of protein were separated using sodium dodecyl sulphate–polyacrylamide gel electrophoresis (Bio-Rad, USA) and electroblotted with antibody against MUC1 (ab45167, Abcam, UK)(1:1000 dilution), NF-κB (ab16502, Abcam, UK)(1:1000 dilution), IκBα (ab76429, Abcam, UK)(1:1000 dilution), phosphorylated IκBα (sc-52943, Santa Cruz Biotechnology, USA)(1:500 dilution), histone H3 (9715, Cell Signaling, USA)(1:1000 dilution) or β-tubulin (Zms1190, Sigma-Aldrich, USA)(1:2000 dilution) and then probed with the corresponding peroxidase-conjugated secondary antibody(Horseradish enzyme labeled goat anti rabbit IgG (H + L) or Horseradish enzyme labeled goat anti mouse IgG (H + L))(ZB-2301, ZB-2305,ZSGB-BIO, China)(all 1:3000 dilution). The signals of the bound antibodies were detected using the Immun-Star HRP Chemiluminescent kit (Bio-Rad, USA).

### RNA extraction and quantitative real-time PCR

Total RNA was extracted from the cultured cells using TRIzol reagent (Invitrogen, USA), according to the manufacturer's instructions. cDNA was reverse transcribed from 1000 ng of total RNA, using the PrimeScript RT reagent kit (TaKaRa), according to the manufacturer's instructions. Real-time PCR was performed using the following primer pairs and the QuantiTect SYBR Green kit (Qiagen, GER), according to the manufacturer's protocol. The MUC1 primers were F- ‘CCTACCATCCTATGAGCGAGTAC’ and R- ‘GCTGGGTTTGTGTAAGAGAGGC’. The GAPDH primers were F- ‘GTCTCCTCTGACTTCAACAGCG’ and R- ‘ACCACCCTGTTGCTGTAGCCAA’.The Real-time PCR was performed on ABI PRISM 7900 HT system (Applied Biosystems, Foster City, CA). The qPCR amplification program was 95 ℃ for 2 min, 95 ℃ for 5 s, and 60 ℃ for 30 s, and the last two steps undergo 40 cycles. For relative quantification, mRNA levles of human MUC1 levels were normalized against GAPDH.

### Luciferase activity assay

A 2910-bp (− 2893 to + 117) segment at the 5′-flanking region of the human MUC1 gene was amplified using PCR. Specific primers from the human MUC1 gene (NC_000001.11) were used: (forward/KpnI) 5′-GGTACCCCAGGAAAGATGACAGCACA-3′ and (reverse/HindIII) 5′-AAGCTTCAGAAAGACCACGAAGACCA-3′. The PCR-amplified DNA of the MUC1 promoter was then cloned into the KpnI/HindIII site of the vector pGL4-Basic (Promega, USA), which contains a Renilla luciferase gene. The PCR products (pGL4-MUC1) were confirmed by electrophoresis and sequencing. The pGL4-MUC1 plasmid was transiently transfected into A549 cells using Lipofectamine 3000 (Invitrogen, USA), according to the manufacturer's instructions. After various treatments, the A549 cells were collected and disrupted, and their Renilla luciferase activity was measured using a luciferase assay system (Promega, USA). The promoter activity was determined by normalising the Renilla luciferase activity to the firefly luciferase activity and was presented in arbitrary units.

## Statistical analysis

GraphPad Prism 5.1 software (GraphPad software, San Diego California, USA) was used for data analysis. The experiments were performed in triplicates and analysed using a two-tailed Student’s t-test or one-way ANOVA. The data are presented as the mean ± standard error of the mean. *P* < 0.01 and *P* < 0.001 were considered significant and extremely significant differences, respectively.

## Results

### Protein levels of MUC1, IL-6, IL-1β, IL-8 and TNF-α were all increased in the sputum of children with RSV bronchiolitis during the exacerbating phase

**Fig. 1:**
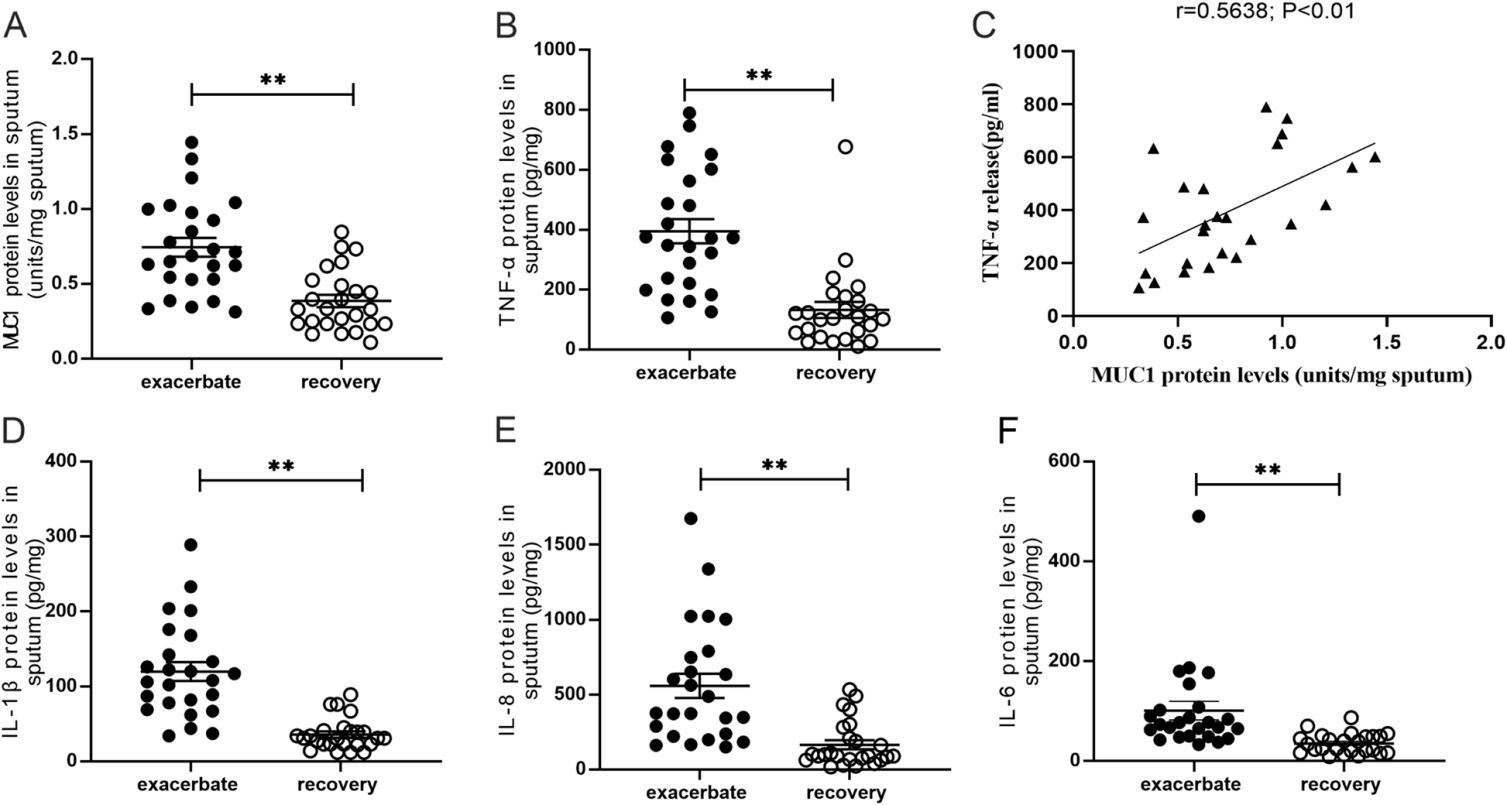
The levels of MUC1 and pro-inflammatory factors in the sputum samples of children with RSV-infected bronchiolitis. The MUCl, TNF-α, IL-1β, IL-8 and IL-6 protein levels in the sputum were determined using ELISA (**A**-**B**, **D**-**F**). All values are expressed as the mean ± standard error of the mean (SEM) (** *P* < 0.01). The relationship between MUC1 and TNF-α protein levels is shown (C, *r* = 0.5638, *P* < 0.01)

Deep sputum samples were collected from children with bronchiolitis infected with primary RSV, and the levels of MUC1 and pro-inflammatory factors were evaluated. The MUC1 protein levels in sputum samples were significantly higher during the exacerbation period than during the recovery period; similar results were observed in terms of the protein levels of TNF-α, IL-6, IL-1β and IL-8 (Fig. 1A-B, D-F). The protein levels of MUC1 had a positive relationship with those of TNF-α (Fig. 1C), but not those of IL-1β, IL-6 and IL-8.

### RSV and TNF-α both induced MUC1 expression in A549 cells

**Fig. 2:**
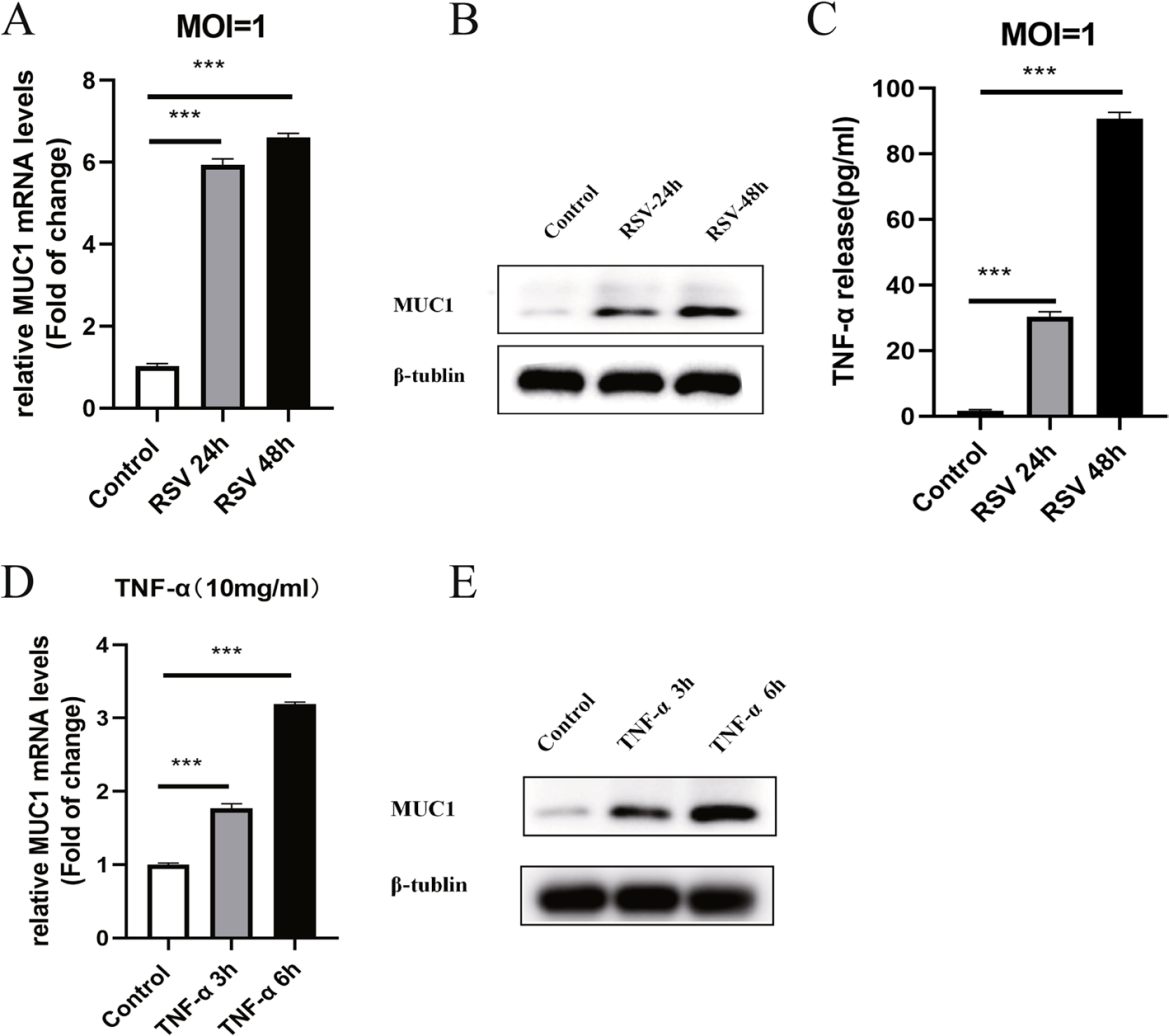
RSV and TNF-α both induced MUC1 over-expression in A549 cells. After RSV infected A549 cells, relative MUC1 mRNA and protein levels were measured by real-time RT-PCR and western blotting at 24 and 48 h time points (**A**, **B**). The TNF-α protein levels after RSV infection were measured using a TNF-α ELISA kit (**C**). The mRNA and protein levels of MUC1 in the A549 cells after TNF-α administration were measured using real-time RT-PCR and western blotting, respectively (**D**, **E**). The data were obtained from three triplicate experiments and shown as the mean ± SEM (***, *P* < 0.001)

The A549 cells expressed the lowest levels of MUC1 under normal conditions. After treatment with RSV (MOI = 1) for 24 h, MUC1 expression and TNF-α release were dramatically increased (Fig. 2A-C,). Treatment with TNF-α (10 ng/ml) increased the expression of MUC1 mRNA in the A549 cells in a time-dependent manner (0, 3 and 6 h) (Fig. 2D). Western blotting confirmed that the MUC1 protein expression was also up-regulated upon TNF-α treatment (Fig. 2E).

### NF-κB inhibition reduced the MUC1 levels induced by RSV and TNF-α in A549 cells

**Fig. 3:**
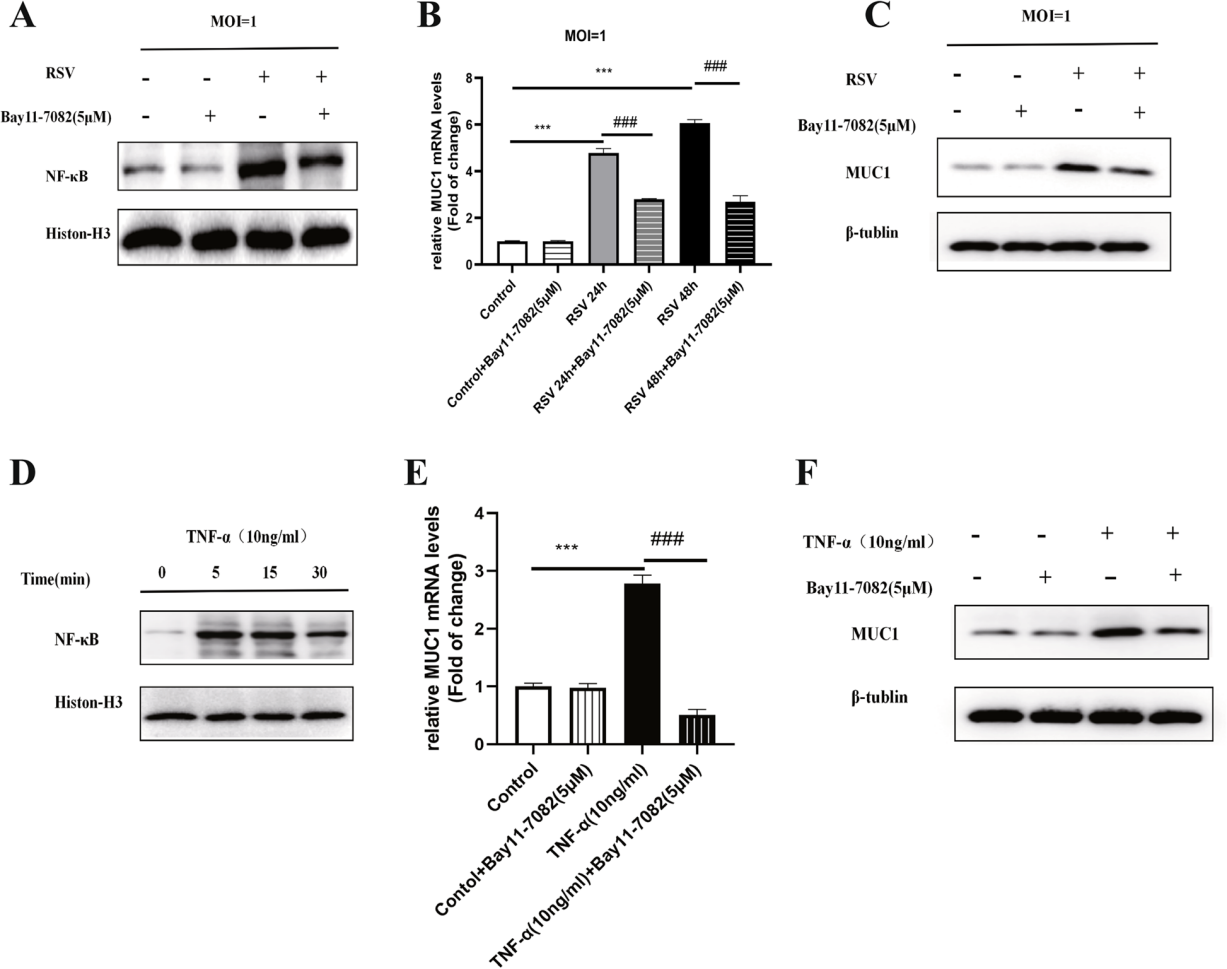
The role of NF-κB in the MUC1 levels induced by RSV and TNF-α in A549 cells. After RSV infected A549 cells, with/without Bay11-7082 treatment, the NF-κB levels in the nuclear extracts at 24 h and were determined by western blotting (**A**). The mRNA levels of MUC1 were measured using RT-PCR at 24 and 48 h after RSV infection and with/without Bay11-7082 treatment (**B**). The protein levels of MUC1 were measured using western blotting at 24 h after RSV infection and with/without Bay11-7082 treatment (**C**). The nucleic NF-κB protein levels at various time points after TNF-α treatment were determined using western blotting (**D**). MUC1 relative mRNA levels and protein levels were detected in the samples after TNF-α administration and with/without Bay 11–7082 treatment (**E**, **F**). The data were obtained from three triplicate experiments and shown as the mean ± SEM (***, *P* < 0.001, control group compared with RSV or TNF-α group; ^###^, *P* < 0.001, RSV group compared with RSV + Bay 11–7082 group or TNF-α group compared with TNF-α + Bay 11–7082 group)

RSV increased NF-κB activation, as shown by the enhancement of its nuclear translocation in airway epithelial cells (Fig. 3A). However, this activation was blocked by pre-treatment with Bay11-7082, a NF-κB inhibitor (Fig. 3A). Using Bay11-7082 significantly reduced the MUC1 mRNA and protein levels in the RSV-infected A459 cells (Fig. 3B and C). TNF-α activated NF-κB signalling by enhancing its nuclear translocation in airway epithelial cells (Fig. 3D). To determine the involvement of NF-κB in TNF-α-induced MUC1 expression, the A549 cells were treated with the NF-κB inhibitor Bay11-7082 after TNF-α administration. Consequently, NF-κB inhibition abolished TNF-α-induced MUC1 expression at the mRNA and protein levels (Fig. 3E and F).

These results suggest that MUC1 over-expression induced by TNF-α and RSV were all regulated by the transcription factor NF-κB in airway epithelial cells.

### The cAMP/PKA pathway may play a role in TNF-α or RSV-induced MUC1 expression through the NF-κB pathway in A549 cells

**Fig. 4:**
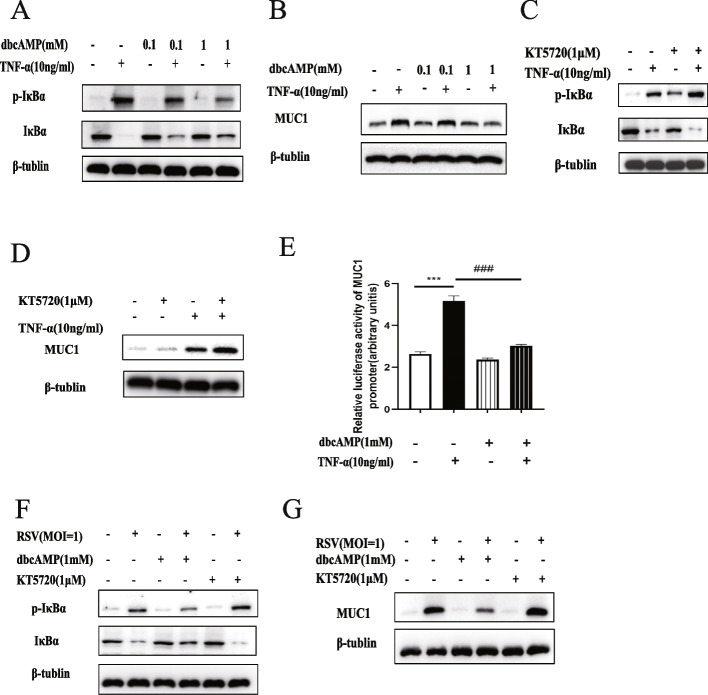
The role of the cAMP/PKA signalling pathway in TNF-α-induced MUC1 up-regulation in A549 cells. The protein levels of IκB, phosphorylated IκB, MUC1 and β-tubulin in the cells after TNF-α administration and with/without dbcAMP treatment were determined using western blotting (**A**,**B**); the total protein levels of IκB, phosphorylated IκB, MUC1 and β-tubulin in cells after TNF-α administration and with/without KT5720 treatment were determined using western blotting (**C**, **D**); the activation levels of MUC1 promoter after TNF-α administration and with/without dbcAMP treatment were determined using the luciferase activity assay (**E**); the total protein levels of IκB, phosphorylated IκB, MUC1 and β-tubulin in the cells after RSV infection and with/without KT5720 or dbcAMP treatment were determined using western blotting (**F**, **G**). The data were obtained from three triplicate experiments and shown as the mean ± SEM (***, *P* < 0.001, control group compared with the TNF-α group; ###, *P* < 0.001, TNF-α group compared with the TNF-α + dbcAMP group)

The vital role of TNF-α in MUC1 over-expression in A549 cells during RSV infection is well known. To explore the relationship between the cAMP/PKA and NF-κB pathways on the modulation of TNF-α or RSV-induced MUC1 up-regulation, we determined the protein levels of MUC1 and phosphorylated protein levels of IκBα using a cAMP analogue (dbcAMP) or a PKA inhibitor (KT5720). Using dbcAMP downregulated the protein levels of p-IκBα and MUC1 in the TNF-α-treated A549 cells (Fig. 4A, B). By contrast, using a PKA inhibitor up-regulated these protein levels, as shown in Fig. 4C and D. The luciferase activity assay results also validated that the MUC1 promoter was activated by TNF-α treatment. Notably, as expected, the MUC1 promoter activation was abolished by dbcAMP (Fig. 4E). In RSV-infected A549 cells, we found that dbcAMP deregulated the MUC1 and p-IκBα protein levels, whereas KT5720 had the opposite effects (Fig. 4F-G).

## Discussion

In this study, we primarily found that the MUC1 protein levels, as well as TNF-α levels, were significantly up-regulated in the sputum of children with RSV-infected bronchiolitis during the exacerbation period. We also validated that NF-κB activity inhibition significantly restrained the up-regulation of MUC1 induced by RSV or TNF-α in A549 cells. Moreover, the cAMP/PKA pathway was found to may play a role in the up-regulation of MUC1 induced by TNF-α or RSV through the p-IκBα/NF-κB signalling pathway. These results revealed the relationship between the pro-inflammatory factor TNF-α and MUC1 over-expression after RSV infection.

Because MUC1 is used as a diagnostic marker for many severe respiratory diseases [6], we first found that the MUC1 protein levels were significantly up-regulated in the sputum of children with RSV-infected bronchiolitis during the exacerbation period; the levels of pro-inflammatory factors, including TNF-α, IL-1β, IL-8 and IL-6 (Fig. 1), were also increased, indicating that higher the pro-inflammatory factor levels, higher are the MUC1 protein levels. Consistent with our results, Zheng Z et al. found that the MUC1 levels are increased in the sputum, along with higher levels of cytokines, such as TNF-α and IL-8, during the acute phase than during the remission phase in patients with chronic obstructive pulmonary disease [11]. Given the protective role of MUC1 during infection, the high levels of the inflammatory cytokines may have induced higher MUC1 expression from epithelial cells to limit RSV-induced progressive inflammation. In addition, Lu W et al. found that the MUC1 protein levels were increased in the sputum of patients with COVID-19, as compared with healthy controls, perhaps due to the presence of detached and disrupted epithelial cells [20]. Because RSV can induce the shedding of infected epithelial cells and cause acute obstruction of the host distal airway [21], the increased levels of MUC1 in the RSV-infected patient sputum samples may be attributable to the shedding of RSV-infected epithelial cells.

To explore the molecular mechanisms underlying the up-regulation of MUC1 levels after TNF-α-induced RSV infection, we inhibited NF-κB activity to detect the MUC1 levels in RSV-infected A549 cells. We found that NF-κB inhibition significantly reduced the mRNA and protein levels of MUC1 induced by RSV and TNF-α in the A549 cells (Fig. 3). Consistent with our results, Xiang S et al. found that crosstalk occurs between NF-κB and lncRNAs (HOTAIR) for mediating MUC1 expression and that NF-κB inhibition can suppress MUC1 expression in castration-resistant prostate cancer cells [22]. Meanwhile, the cAMP/PKA pathway is known to regulate mucus expression. For example, the expression of MUC2, which plays a protective role in the gut [23], is induced by vasoactive intestinal peptide, which is partly dependent on the cAMP/PKA pathway [24]. Moreover, *S. pneumoniae* can induce MUC5AC protein levels partly by activating the cAMP/PKA pathway in human middle ear epithelial cells [25]. However, Zhu T et al. found that glucagon-like peptide-1 (GLP-1) reduced the MUC5AC mRNA and protein levels by activating PKA activity and inhibiting NF-κB activation in mice with ovalbumin-induced asthma [26]. This indicates that cAMP/PKA signalling may play different roles in mucin expression in different situations. Our study validated that TNF-α and RSV both induced MUC1 expression and NF-κB activation in A549 cells; both these processes were enhanced by PKA inhibition, reduced by cAMP analogue (Fig. 4). Considering TNF-α is the key regulator of MUC1 during RSV infection, the cAMP/PKA pathway may regulate MUC1 expression through NF-κB signalling during RSV infection. Moreover, we found that cAMP can significantly suppress the activation of MUC1 promoter (Fig. 4F). Lagow EL et al. reported that the stimulation of MUC1 expression by TNF-α in normal human mammary epithelial cells was dependent on the binding of NF-κB p65 to the MUC1-κB site (− 589/ − 580) [27]. Thus, the cAMP/PKA signalling pathway may induce MUC1 expression through NF-κB activation through the high TNF-α protein levels induced by RSV infection. Moreover, Koga T et al. found that TNF-α induces MUC1 gene transcription in A549 cells through the Sp1 binding in the MUC1 promoter located at site SP1 (− 99/ − 90) [14]. We will further investigate whether there exists a correlation between SP1 and NF-κB in inducing MUC1 expression after RSV infection.

In summary, we found that MUC1 protein levels were significantly increased in the sputum of children with RSV bronchiolitis during the exacerbating phase, indicating that MUC1 may be positively associated with the progression of RSV infection. Furthermore, we found that the cAMP-PKA-NF-κB pathway regulates the MUC-1 protein levels induced by TNF-α during RSV infection in A549 cells.

### Supplementary Information

**Additional file 1.** 

**Additional file 2.**

## Data Availability

The datasets are not publicly available due to patients confidentiality but are available from the corresponding author on reasonable request.
