# Supplementary material for: Role of the cAMP-PKA-NF-κB pathway in Mucin1 over-expression in A549 cells during Respiratory syncytial virus infection
Source: BMC Infect Dis. 2023 Nov 30;23:845. doi: 10.1186/s12879-023-08837-1 (PMC10687811; doi:10.1186/s12879-023-08837-1)
Supplement: Supplementary file 1 — Additional file 1. [file 12879_2023_8837_MOESM1_ESM.pdf]

Methods：

1、Cell viability assay

The viability of the A549 cells was detected using 3-(4,5-dimethylthiazol-2-yl)-2,5-diphenyl-2H-tetrazolium bromide (MTT) assay (KGA311, Keygen Biotech, China), according to the manufacturer's protocol.

2、Immunofluorescence staining

The A549 cells were exposed to sucrose-cushioned purified RSV (MOI = 1) . After 48 h of infecting, the infected A549 cells were fixed for 48 min with 4% paraformaldehyde and subsequently permeabilised with PBS containing 0.5% Triton-100 for 15 minutes and finally blocked with 5% BSA blocking solution for 1 h. The cells were incubated with an anti-RSV primary antibody (1:100 dilution) (Thermo Fisher Scientific, USA) at 4°C, followed by incubation with an Alexa Fluor 647 donkey anti-goat IgG antibody (1:200 dilution) (Thermo Fisher Scientific, USA) for 1 h and 4',6-diamidino-2-phenylindole for 5 min; the cells were then washed with PBS five times for 5 min, and the cell fluorescence intensity was assessed using fluorescence microscopy.

3、Statistical analysis

GraphPad Prism 5.1 software (GraphPad software, San Diego California, USA) was used for data analysis. The experiments were performed in triplicates and analysed using a two-tailed Student's t-test. The relationship between the MUC1 and IL-6, IL-8, IL-1β protein levels was assessed using Person's correlation coefficient analysis.

Figures:

Figure 1

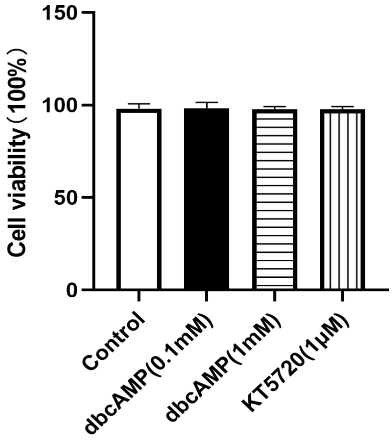

The Cell viability of the A549 cells treated with dbcAMP or KT5720 detected by using the MTT assay

Figure 2

A B C

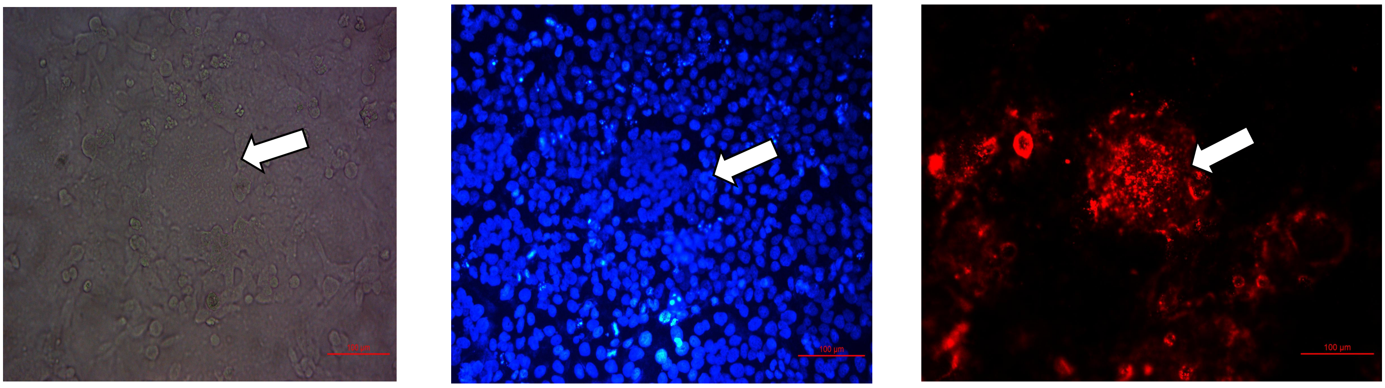

The Immunofluorescence staining of RSV primary antibody in A549 cells after infection

A, Cell state under normal light; B, the cell nuclei counterstained with DAPI (blue); C, the cell stained with the RSV antibody (red)

The white arrow points to the formation of RSV-induced large syncytia.

Figure3

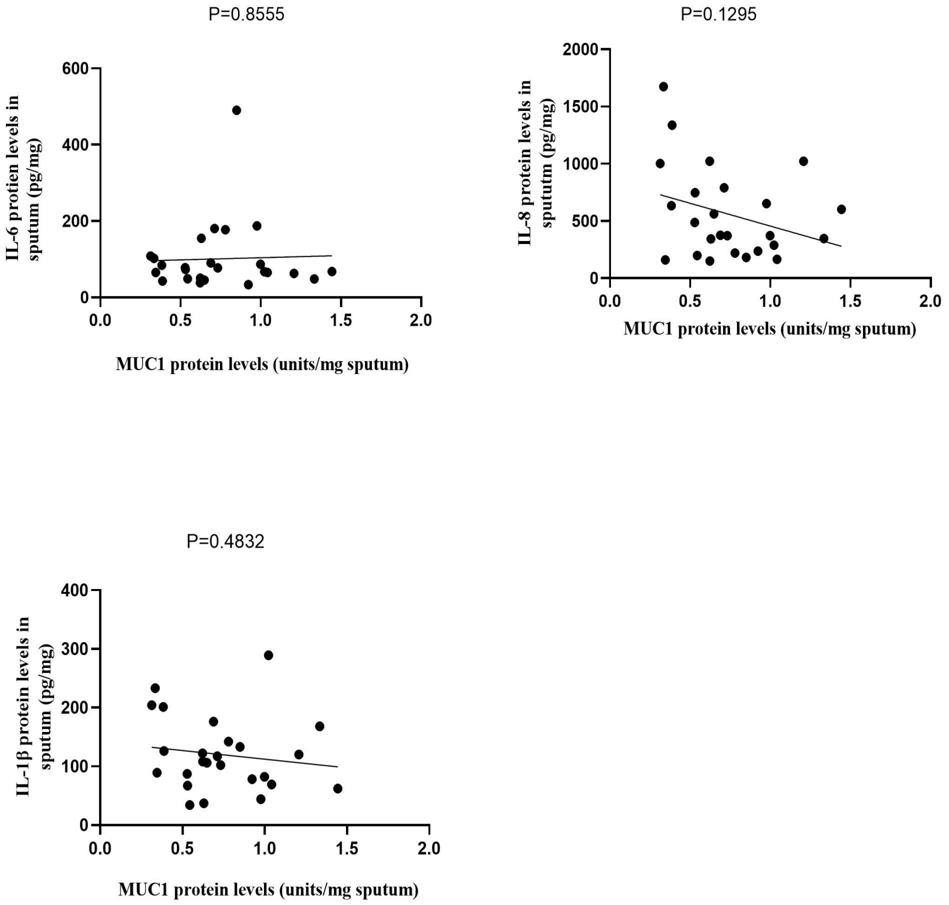

the relationship between the MUC1 protein levels and IL-6, IL-8, and IL-1β protein levels
