# Supplementary material for: Role of the cAMP-PKA-NF-κB pathway in Mucin1 over-expression in A549 cells during Respiratory syncytial virus infection
Source: BMC Infect Dis. 2023 Nov 30;23:845. doi: 10.1186/s12879-023-08837-1 (PMC10687811; doi:10.1186/s12879-023-08837-1)
Supplement: Supplementary file 2 — Additional file 2. [file 12879_2023_8837_MOESM2_ESM.pdf]

Figure2B

MUC1

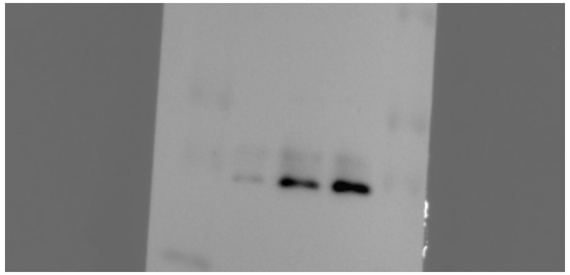

β-tublin

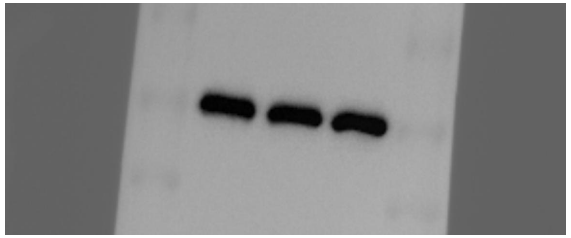

Figure2E

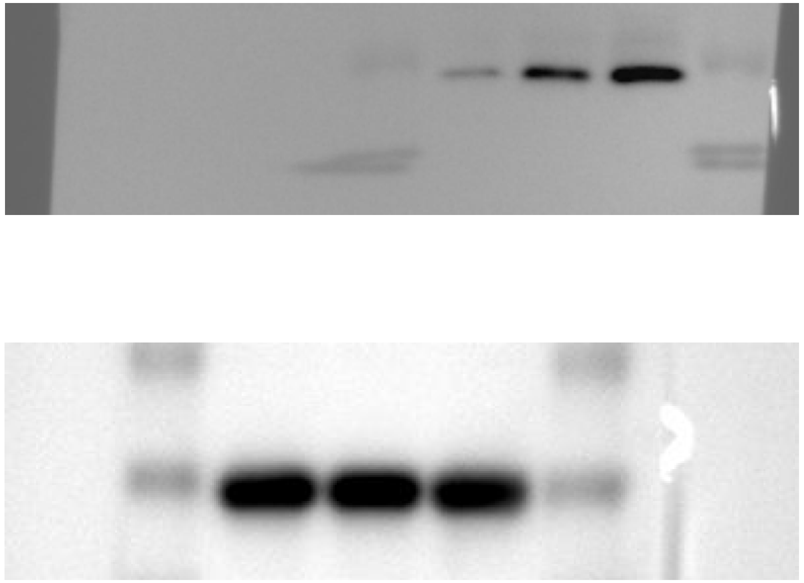

MUC1

β-tublin

Figure3A

NF-κB

Histon-H3

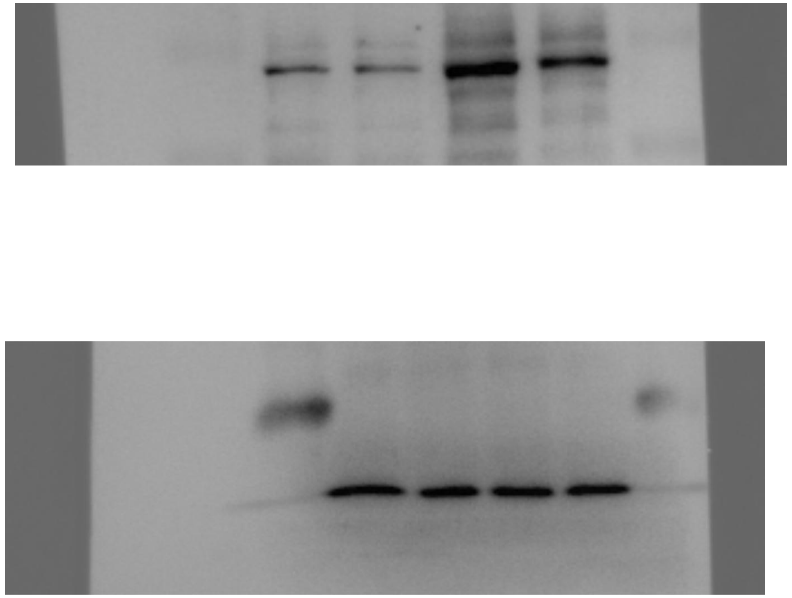

Figure3C

MUC1

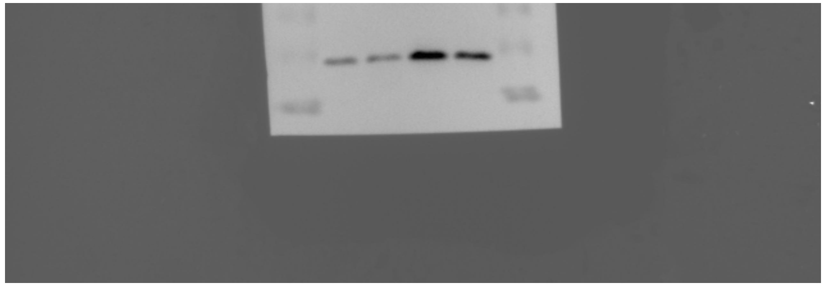

β-tublin

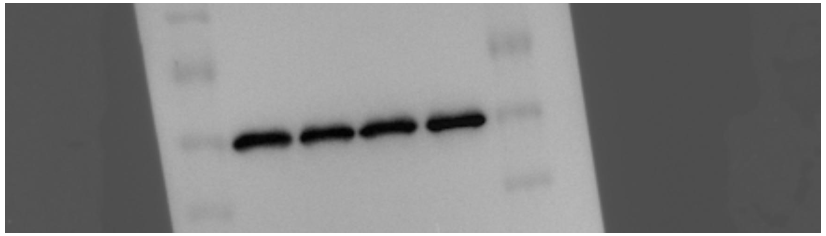

Figure3D

NF-κB

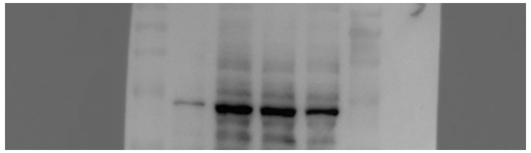

Histon-H3

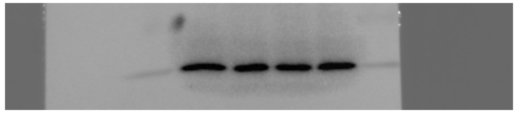

Figure3F

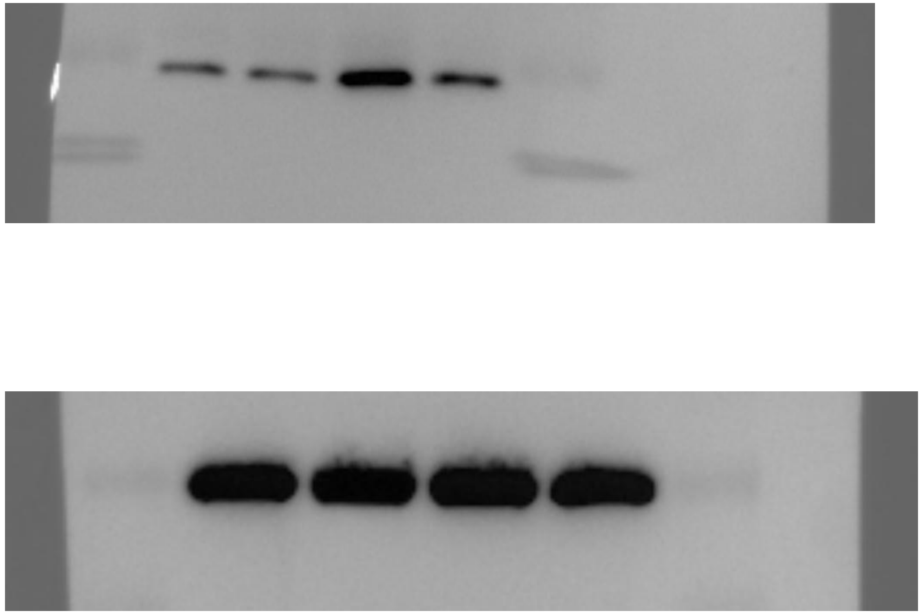

Figure4A

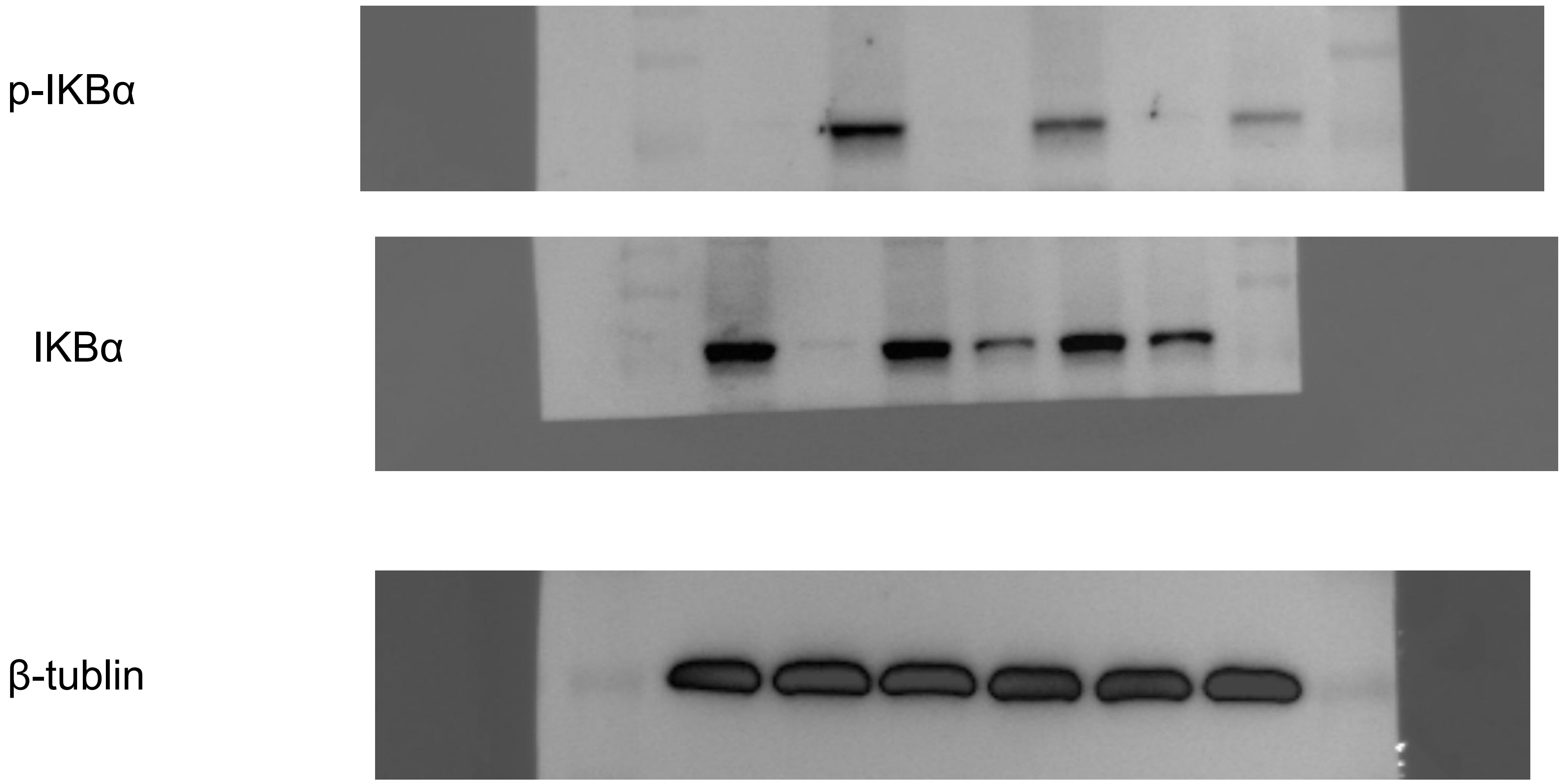

Figure4B

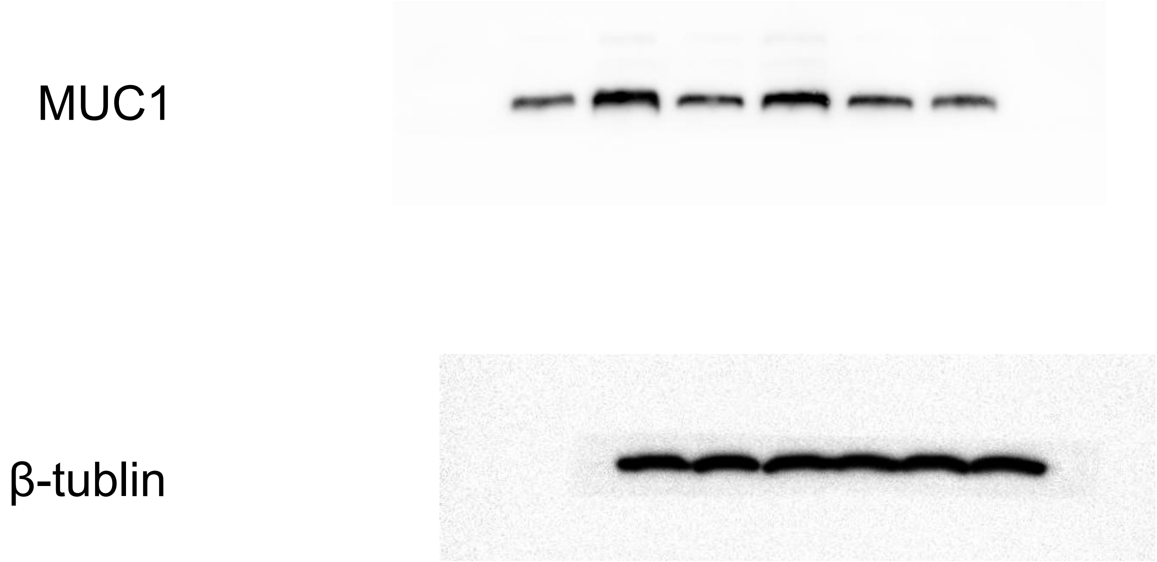

Figure4C

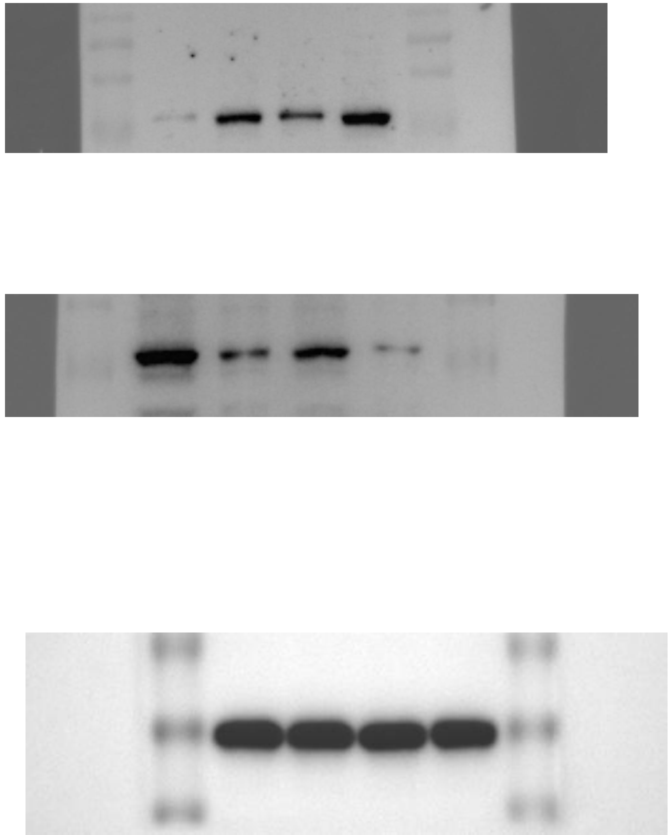

p-IKBα

IKBα

β-tublin

Figure4D

MUC1

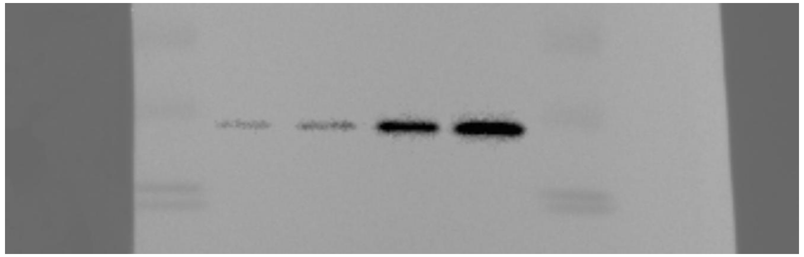

β-tublin

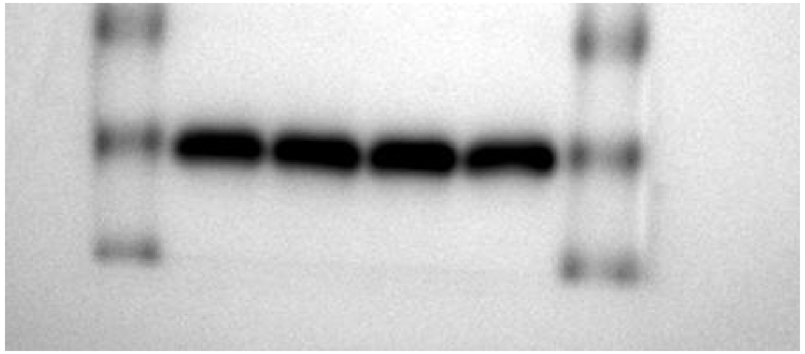

Figure4F

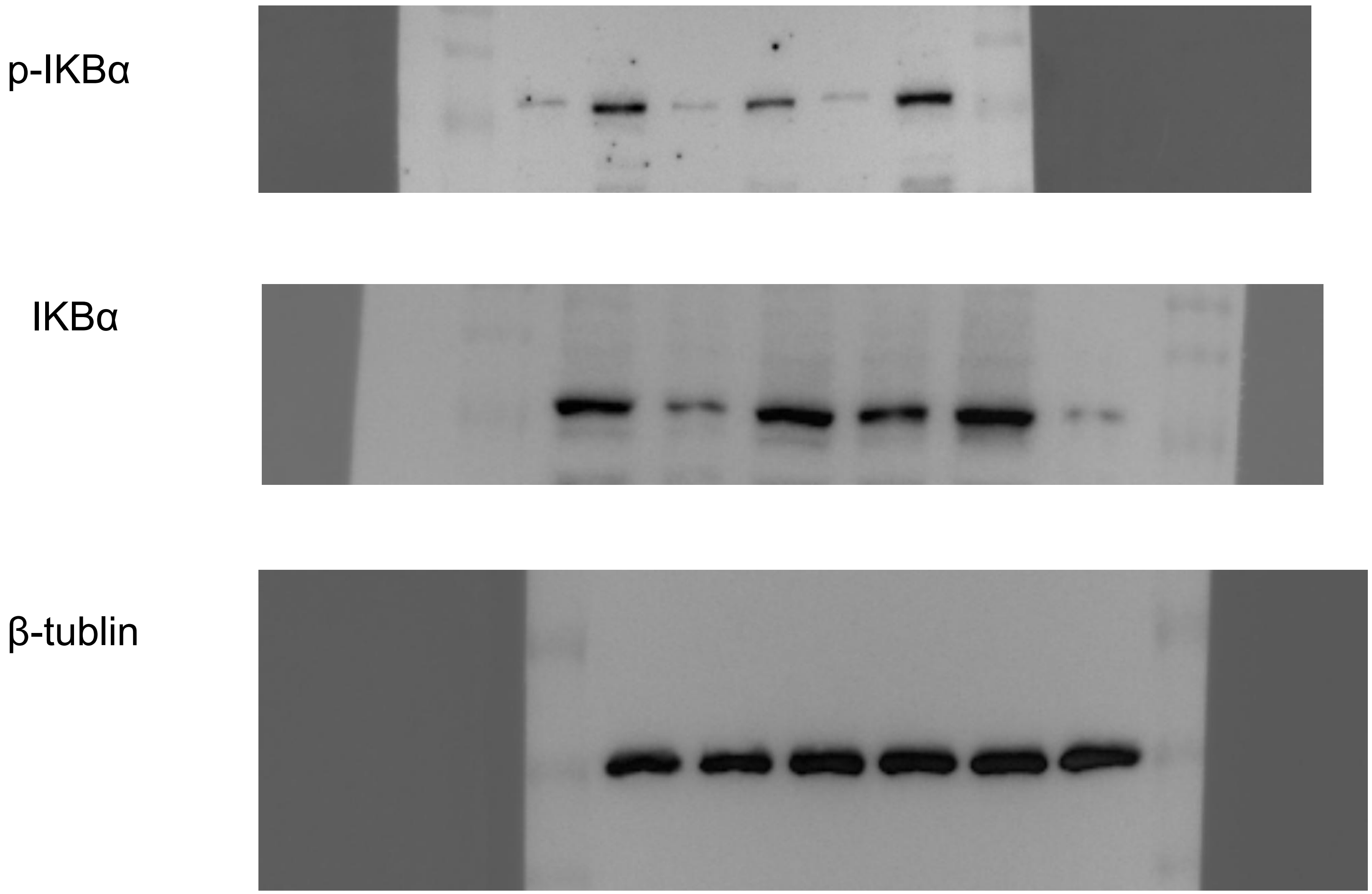

Figure4G

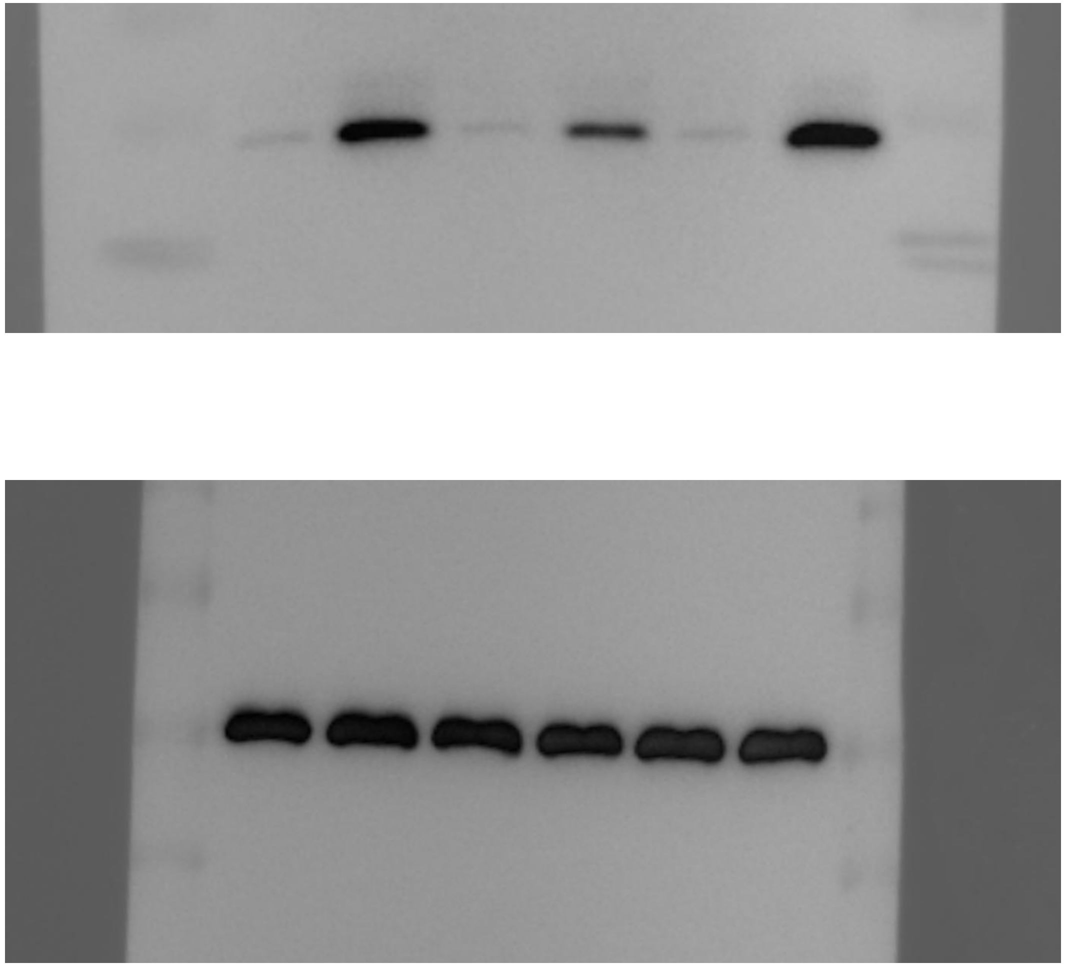

MUC1

β-tublin
